# Effect of a Simulation-Based Teaching Module on Intravenous Drug Administration Among Phase II MBBS Students in Pharmacology: A Competency-Based Medical Education Initiative

**DOI:** 10.7759/cureus.84667

**Published:** 2025-05-23

**Authors:** Aditi N Patil, Madhushree S Magadum, Ramchandra P Limaye, Santoshkumar A Shetti, Shabbir R Pendhari, Sachin G Jagdale

**Affiliations:** 1 Pharmacology and Therapeutics, Bharati Vidyapeeth (Deemed to be University) Medical College and Hospital, Sangli, IND; 2 Pharmacology and Therapeutics, Jagadguru Gangadhar Mahaswamigalu Moorsavirmath Medical College, Hubballi, IND; 3 Pharmacology and Therapeutics, Prakash Institute of Medical Sciences and Research, Urun-Islampur, IND

**Keywords:** cbme, communication skills, intravenous drug administration, mannequin, pharmacology

## Abstract

Background

Pharmacology lectures focus on the knowledge domain, teaching various oral and parenteral routes of drug administration. Before the implementation of competency-based medical education (CBME), practical sessions aimed to enhance students’ understanding of drug administration routes. However, after the CBME curriculum, soft and psychomotor skills are also being given importance. Demonstration of the correct drug administration method through the intravenous route minimizes errors in drug administration and helps in the early acquisition of knowledge and skills. Our study aimed to evaluate the effect of a simulation-based teaching module on intravenous drug administration and communication skills in pharmacology according to the CBME curriculum.

Methodology

This cross-sectional study was conducted among Phase II students in the Department of Pharmacology, Bharati Vidyapeeth (Deemed to be University), Medical College and Hospital, Sangli, Maharashtra, India, over three years. The teaching faculty first showed videos of intravenous drug administration to the students, followed by demonstrations on mannequins. We collected students’ perceptions at the end of the session. Using the Objective Structured Practical Examination (OSPE) checklist, we also assessed students’ practical skills of intravenous drug administration on mannequins.

Results

In total, 406 students participated in our study. Overall, 89.4% of students felt that learning the intravenous route of drug administration correctly on a mannequin would help in managing various patients. In total, 50% of students were evaluated through OSPE using a checklist. The students were found to be good at intravenous drug administration on the mannequin. Overall, 69% of students communicated about the procedure and alleviated apprehension. Moreover, 97.1% of students sterilized their hands before administering injections. Finally, 28.10% of students were unable to load the syringe properly.

Conclusions

The simulation-based teaching module on intravenous drug administration introduced by the CBME curriculum in practical classes was well accepted by students, who felt more confident in the injection technique on the mannequin. However, a few students needed more practice sessions. During practice sessions, the steps of intravenous injection, such as communicating with the patient before and after injection, applying and removing the tourniquet, and inserting the needle at the proper angle, should be emphasized.

## Introduction

In the last few years, before the competency-based medical education (CBME) curriculum, MBBS teaching for pharmacology changed significantly after stopping animal experiments for undergraduates. Didactic lectures were the primary mode of teaching. The practical aspects of the teaching included tasks such as writing prescriptions, fixed-dose combinations, and dosage formulation. These tasks focus solely on knowledge acquisition and do not lead to the development of psychomotor skills. Thus, students acquire excellent knowledge but lack psychomotor skills, such as the ability to administer drugs through various routes, communicate prescriptions, communicate procedures they wish to perform, and fill out adverse drug reaction forms, among other things [1].

The United States National Coordinating Council for Medication Error Reporting and Prevention defines a medication error as “any preventable event that may cause or lead to inappropriate medication use or patient harm while the medication is in the control of the health care professional, patient, or consumer.” Medication administration error is one of the most common errors in the medication error process [2]. Medication errors are of different types, such as knowledge-based, rule-based, action-based, or memory-based. Medication administration errors are action-based errors, resulting in incorrect outcomes due to imperfect action execution. Training can prevent technical or action-based errors [3]. Compared to other medication administrations, intravenous drug administrations are associated with a higher risk and severity of error. A considerable proportion of errors suggest skill and knowledge deficiencies, with mistakes and severity reducing as clinical experience increases [4]. The unsafe practices used in injection techniques by healthcare workers may include reusing a syringe for more than one patient, double dipping of vials, reusing single-use medication, and not following aseptic precautions or basic injection safety measures. It can lead to profound consequences such as bleeding, bacterial infection, and transmission of blood-borne diseases such as hepatitis B, hepatitis C, and HIV [5-7].

Kuitunen et al. [8] observed that unsafe use of high-alert medications, lack of knowledge of the drug, dose calculation, failure to double-check details of procedures, and confusion between look-alike and sound-alike drugs are the most common causes of errors in intravenous drug administration. Hence, there is a need to focus on medication safety activities related to prescribing, preparation, and administration of intravenous medications. Therefore, training medical students in injection techniques is important to decrease errors in intravenous drug administration.

Students also feel anxious while administering the injection directly to the patient without any prior practice. Learning this skill on a mannequin will improve their confidence and decrease the anxiety about administering the injection to the patient. This activity will make students more engaged in the practical sessions [9].

In India, CBME replaced traditional medical education in 2019. CBME has included various skill-related competencies in pharmacology, e.g., administration of drugs through various routes using mannequins [10-12]. Additionally, it incorporates assessments that verify the attainment of knowledge and skill. The Objective Structured Practical Examination (OSPE) serves as a structured method for evaluating students’ competence [13]. To assess the practical skill in pharmacology, OSPE serves as a relevant, meaningful, and feasible tool. It also decreases examiners’ bias [14].

CBME aims to prepare medical students who can perform intravenous drug administration independently. This will increase their confidence and decrease medication errors while administering injections to actual patients during the later phase of MBBS and internship. This will also enhance their competency, leading to successful clinical practice and ensuring community safety by avoiding untoward complications. Hence, we aimed to study how Phase II MBBS students perceive the new simulation-based teaching module and to evaluate the skills they learned about drug administration by intravenous route on mannequins. This study aimed to evaluate the effect of a simulation-based teaching module on intravenous drug administration and communication skills in pharmacology according to the CBME curriculum.

## Materials and methods

This study was approved by the Institutional Ethics Committee, Bharati Vidyapeeth (Deemed to be University), Medical College and Hospital, Sangli (approval number: BV(DU)MC&H/Sangli/IEC/441/21).

This cross-sectional study was conducted in the Department of Pharmacology, Bharati Vidyapeeth (Deemed to be University), Medical College and Hospital, Sangli. The study involved students who attended the practical teaching hours conducted as per the competency-based curriculum. Prior sensitization of the simulation-based teaching module and its importance was done. Written informed consent was taken before the study.

In a practical batch, students were divided into five small groups of 10 each. These groups were allotted to five facilitators. The students received detailed information on intravenous drug administration. A video on intravenous drug administration was shown to the students. Demonstration of correct intravenous drug administration was conducted on mannequins to cover pharmacology competency PH 4.1. This included information on material required, safety concerns, related communication with the patient and privacy considerations, and detailed procedures (identification of the injectable site, site preparation, and process of injection with all aseptic precautions). It also included aspiration of the drug from a vial or ampoule (after proper breaking technique) and dissolution of the dry medicine. Knowledge of adverse effects and patient responses was taught during practical sessions. Each group was given two hours to practice these skills on mannequins. They were provided with feedback questionnaires (Table 1) to fill in their perceptions about the new teaching techniques. It consisted of a total of 10 questions, of which nine were based on a five-point Likert scale. The scale was rated as strongly disagree, disagree, neutral, agree, and strongly agree. The questionnaire underwent validation and reliability tests. The reliability test revealed a Cronbach’s alpha value greater than 0.7, indicating good internal reliability. The last question was open-ended.

**Table 1:** Questionnaire to assess students’ perception of the teaching module on intravenous drug administration and related communication skills. IV: Intravenous

Questions
Learning intravenous drug administration is relevant to future practice
The teaching technique on intravenous injection skills was well-planned and coordinated
I am more confident about intravenous drug administration in the future
After the sessions, I feel better equipped to communicate with the patient
Intravenous drug administration should be taught to all medical students
Teaching sessions increased my interaction with my teachers
Teaching methods were beneficial in learning the practical skills
I found the teaching sessions on skills interesting
The time allocated was adequate
What do you think could be done differently?

In addition to the above, a predesigned, validated, structured checklist for intravenous drug administration was prepared, and stations for OSPE (Table 2) were set up in the next class [15]. We randomly selected 50% of students and asked them to perform intravenous drug administration. We evaluated the students at different steps.

**Table 2:** Objective Structured Practical Examination (OSPE) checklist for the intravenous injection technique on mannequins.

Checklist
Check the patient’s details and prescription
Communicate with the patient regarding the procedure you are going to perform and allay their apprehensions, if any
Perform hand hygiene
Aspiration of the drug from the ampoule/vial
Uncover the arm to be injected, apply the tourniquet, and stabilize the vein with the non-dominant hand
Clean the injecting site with an alcohol swab in a firm circular motion
Insert the needle at a 25–30° angle
Aspirate briefly before the injection to check for blood aspirate
Inject the drug slowly (0.5–2-minute time interval) after loosening the tourniquet
Evaluate the patient for any immediate side effects, locally and systemically

The minimum sample size, calculated using the following statistical formula: n = (Z² × p × (1-p))/d², was 346. The confidence level (1-α) was 99% with absolute precision (d) 6%, and p (the proportion of students feeling more confident in intravenous drug administration in the future) was 75% [1]. We then employed convenience sampling. Accordingly, the study involved all Phase II MBBS students from 2021 to 2023 to meet the sample size. Therefore, 406 students participated in the study.

The data obtained were entered into a Microsoft Excel (Microsoft Corp., Redmond, WA, USA) sheet, and statistical analyses were performed using SPSS Version 22 (IBM Corp., Armonk, NY, USA). Results are presented using descriptive statistics (frequency and percentage).

## Results

A total of 406 students participated in the study. Most students, 363 (89.41%), believed learning intravenous drug administration on a mannequin was relevant to future practice, 308 (75.86%) students strongly agreed that the teaching module used was well planned and coordinated, and 257 (63.30%) students felt more confident in intravenous drug administration. Among 406 students, 276 (67.98%) felt better equipped to communicate with the patients in the future regarding details of the drug, dosage, and its side effects; 346 (85.22%) students strongly agreed that all medical students, across all phases, should receive this teaching session; and 319 (78.57%) students felt that the teaching module helped in improving their interactions with teachers. According to 330 (81.28%) students, this teaching method is beneficial in learning practical skills, 328 (80.79%) students found the session interesting, and 269 (66.26%) students strongly agreed that the time allocated to the session was adequate (Table 3).

**Table 3:** Perception of students toward the teaching module on IV drug administration and communication skills on mannequins. IV = intravenous; n = frequency

Question	Strongly agree		Agree		Neutral		Disagree		Strongly disagree	
	n	%	n	%	n	%	n	%	n	%
Learning IV drug administration is relevant to future practice	363	89.41	40	9.85	2	0.49	0	0.00	1	0.25
The teaching technique on IV skills was well planned and coordinated	308	75.86	91	22.41	5	1.23	2	0.49	0	0.00
I am more confident about IV drug administration in the future	257	63.30	120	29.56	23	5.67	4	0.99	2	0.49
After the sessions, I feel better equipped to communicate with the patient	276	67.98	97	23.89	22	5.42	10	2.46	1	0.25
IV drug administration should be taught to all medical students	346	85.22	54	13.30	2	0.49	3	0.74	1	0.25
Teaching sessions increased my interaction with my teachers	319	78.57	67	16.50	12	2.96	3	0.74	5	1.23
Teaching methods were beneficial in learning the practical skills	330	81.28	70	17.24	4	0.99	1	0.25	1	0.25
Found the teaching sessions on skills interesting	328	80.79	71	17.49	6	1.48	1	0.25	0	0.00
The time allocated was adequate	269	66.26	88	21.67	32	7.88	15	3.69	2	0.49

Most students were satisfied with the session. Responses and suggestions from some students for the open-ended question were as follows: these sessions should be conducted more often, more time should be given for demonstration and practice, it should be shown on real patients, casualty and hospital visits should be included, and each student should get a sufficient amount of time.

Of 406 students, 210 were randomly selected for the OSPE station of intravenous drug administration. During the intravenous drug administration, 42 (20%) students failed to check the prescription order before giving the injection, 145 (69.05%) students communicated the procedure and allayed apprehension, and 204 (97.14%) students sanitized their hands before giving injections. However, 59 (28.10%) students were unable to load the syringe properly (i.e., cleaning of cork, quantity of drug withdrawn from vial, removal of air bubble from the syringe, etc.). In addition, 120 (57.14%) students missed the application of the tourniquet before giving the injection. The majority of the students, 184 (87.62%), sterilized the injection site with an alcohol swab in a firm circular motion before injection, and 81 (38.57%) students were unsuccessful in inserting the needle at the proper angle. The plunger was not pulled back by 76 (36.19%) students to check for blood aspirates. While 77 (36.67%) students injected drugs slowly after loosening the tourniquet, only 64 (30.48%) students evaluated immediate discomfort and adverse effects (Table 4).

**Table 4:** Objective Structured Practical Examination (OSPE) checklist for the intravenous injection technique on mannequins. Yes (n) = number of students who followed the step; No (n) = number of students who did not follow the step

Evaluation criteria	Yes (n)	%	No (n)	%
Safety concern	168	80	42	20
Communication with the patient	145	69.05	65	30.95
Perform hand hygiene	204	97.14	6	2.86
Aspiration of the drug from the vial	151	71.9	59	28.1
Uncover the arm and apply the tourniquet	90	42.86	120	57.14
Sterilization of the injection site	184	87.62	26	12.38
Insert the needle at 25–30 degrees	129	61.43	81	38.57
Aspirate briefly before the injection	134	63.81	76	36.19
Inject the drug slowly after loosening the tourniquet	77	36.67	133	63.33
Evaluation of patient response	64	30.48	146	69.52

## Discussion

We conducted this study considering two objectives: the first was regarding students’ perception of the new simulation-based teaching module, and the second was assessing their skills. In our study, hands-on training of various injection techniques on mannequins was provided to students. It increased the confidence level, understanding, and skill level of students. Practicing on mannequins improved practical skills, but few students reported that it did not give the experience of real patients. In our study, 89% of students found this new simulation-based teaching module relevant to future practice, and 63% of students felt confident in administering intravenous injections to mannequins. Students skipped a few steps throughout the OSPE session, including assessing the patient’s response after giving the injection, applying and removing the tourniquet, positioning the needle at the right angle, and communicating with the patient both before and after the injection.

It has been observed that medication errors can occur during any stage of the treatment process, including drug administration, which can be a significant cause of mortality. Different studies have reported that intravenous drugs are commonly associated with drug administration errors [16,17]. Hence, training medical students during their initial years is important to decrease such medication errors. WHO defines a safe injection as one that does not harm the recipient or expose the healthcare provider to avoidable risk [18]. CBME has included various competencies, such as the administration of drugs through various routes in mannequins. For medical students studying pharmacology, OSPE is a useful and skill-building assessment tool. It assures improved student performance and eliminates prejudice, stress, and repetition in the test. However, there are logistical challenges and considerable planning involved [19].

A study conducted by Khatavkar et al. [20] showed that the confidence level of 84% of students increased after training on the injection technique on mannequins. In the same study, 93.27% and 84.87% of students showed improvement in understanding and skill, respectively, after the session. In a study by Bhanwra et al. [1], 96% of students believed that learning drug administration on a mannequin is relevant to future practice, which is comparable to our findings. In the same study, 95% of students felt better equipped for communication, whereas in our study, students found it difficult to communicate with patients. The students’ perception regarding the module was positive, indicating that the majority of students were satisfied with it. Some students felt that it could be taught on actual patients in wards.

In a study conducted by Panigrahy et al. [21], 10% of students failed to cross-check medicines before administering injections, and 87.5% of students prepared the injection site with antiseptic. In another study conducted by James et al. ​[22], 16% of students failed to double-check the prescription order before the beginning of the procedure, which is comparable to our study findings. In the same study, 90% of students sanitized their hands before handling anything, and 80% of students loaded syringes properly, which is also comparable to our study findings. In our study, students failed in the application of the tourniquet before injection and its removal before administering the injection. They also failed in the evaluation of the immediate adverse effects after administering the injection. It is possible that students forgot the steps of the intravenous injection procedure because they were performing it on mannequins for the first time. Repeated training sessions that emphasize these steps and provide the checklist can help address these inadequacies. It can also be enhanced by providing appropriate clinical relevance and outlining the benefits and drawbacks of each step.

Valentin et al. [23] found that trainees were responsible for 50% of parenteral drug administration errors and needed to be supervised. Training students in parenteral drug administration on mannequins in the second year will reduce their anxiety and improve their skill and confidence when administering injections to real patients. Development of the OSPE checklist will help stress the steps that are missed or wrongly performed by students. It will also help assess students during practical exams. It is a relevant topic in the context of CBME, and we have used an appropriate simulation (mannequin-based) for skill training. We were able to generate student engagement and positive feedback.

Although the study had a sufficient sample size, a multicentric study would have been more generalizable. In our study, as we did not check skill before implementation of the module, we could not compare our results pre- and post-intervention. We could not assess all students who went through the module due to limited infrastructure and time constraints. The OSPE checklist included only the cardinal steps of intravenous drug administration due to time limitations, which could include all steps of intravenous injection. Further studies should be done to evaluate the performance of students on real patients.

## Conclusions

The simulation-based teaching module on intravenous drug administration using mannequins was both practical and well-received by the students. Institutions should provide students with additional practice time through multiple sessions, focusing on the steps they may have missed and emphasizing their importance. A checklist was developed to assess student performance in the OSPE and provide feedback. Further studies involving interns, utilizing pre- and post-intervention analyses, should be conducted at various medical institutions to enhance the generalizability of the findings. Various other medical institutions should conduct such interventional research among interns to have a better understanding and to generalize and adopt the findings.
